# Supplementary material for: Mineral-Solubilizing Bacteria-Mediated Enzymatic Regulation and Nutrient Acquisition Benefit Cotton’s (Gossypium hirsutum L.) Vegetative and Reproductive Growth
Source: Microorganisms. 2023 Mar 28;11(4):861. doi: 10.3390/microorganisms11040861 (PMC10146682; doi:10.3390/microorganisms11040861)
Supplement: Supplementary file 1 [file microorganisms-11-00861-s001.zip › microorganisms-2258498-supplementary.pdf]

Article

Mineral-Solubilizing Bacteria-Mediated Enzymatic Regulation and Nutrient Acquisition Benefit Cotton's (*Gossypium hirsutum* L.) Vegetative and Reproductive Growth

Iqra Ahmad [1], Maqshoof Ahmad [1,*], Bushra [1], Azhar Hussain [1], Muhammad Zahid Mumtaz [2,*], Najm-ul-Seher [1], Ghulam Hassan Abbasi [3], Farheen Nazli [3], Lisa Pataczek [4] and Hayssam M. Ali [5]

[1] Department of Soil Science, The Islamia University of Bahawalpur, Bahawalpur 63100, Pakistan
[2] Institute of Molecular Biology and Biotechnology, The University of Lahore, Lahore 54000, Pakistan
[3] Institute of Agro-Industry and Environment, The Islamia University of Bahawalpur, Bahawalpur 63100, Pakistan
[4] Institute of Landscape and Plant Ecology, University of Hohenheim, Ottilie-Zeller-Weg 2, 70599 Stuttgart, Germany
[5] Department of Botany and Microbiology, College of Science, King Saud University, Riyadh 11451, Saudi Arabia
* Correspondence: maqshoof_ahmad@yahoo.com (M.A.); zahidses@gmail.com (M.Z.M.)

Supplementary data

Supplementary Table S1. Effect of PSB and ZSB strains inoculation on N, P, and K contents in soil.

Treatment	N content in soil (%)	P contents in soil (mg kg^{-1})	K content in soil (mg kg^{-1})
Absolute control	0.023 ± 0.0004 d	5.0 ± 0.10 f	98.3 ± 1.69 e
Recommended NPK	0.024 ± 0.0005 c	5.2 ± 0.08 ef	101.0 ± 1.86 de
B. subtilis IA6	0.025 ± 0.0003 a-c	5.3 ± 0.08 c-e	102.5 ± 1.96 c-e
P. polymyxa IA7	0.025 ± 0.0003 bc	5.3 ± 0.08 de	104.2 ± 1.60 b-d
Bacillus sp. IA16	0.025 ± 0.0003 a-c	5.5 ± 0.10 a-d	103.3 ±1.82 cd
B. aryabhattai IA20	0.025 ± 0.0004 a-c	5.4 ± 0.09 b-d	104.3 ± 1.65 b-d
B. subtilis IA6 + *Bacillus* sp. IA16	0.026 ± 0.0004 ab	5.7 ± 0.10 a	109.5 ± 1.61 a
B. subtilis IA6 + *B. aryabhattai* IA20	0.025 ± 0.0004 a-c	5.6 ± 0.05 ab	108.3 ± 1.65 ab
P. polymyxa IA7 + *Bacillus* sp. IA16	0.025 ± 0.0003 a-c	5.6 ± 0.06 ab	106.3 ± 1.86 a-c
P. polymyxa IA7 + *B. aryabhattai* IA20	0.026 ± 0.0003 a	5.5 ± 0.07 a-c	105.8 ± 1.68 a-d
LSD ($p \leq 0.05$)	0.001	0.2322	
